# COVID-19 lockdown and its impact on mental health in various population groups in Greece: A cross-sectional study

**DOI:** 10.1192/j.eurpsy.2021.1768

**Published:** 2021-08-13

**Authors:** K. Argyropoulos, K. Krikonis, P. Alexopoulos, D. Avramidis, P. Gourzis, E. Jelastopulu

**Affiliations:** 1 Department Of Public Health, Medical School, University of Patras, Patras, Greece; 2 Statistical Analysis, DatAnalysis, Ioannina, Greece; 3 Department Of Psychiatry, School of Medicine, University of Patras, Patras, Greece; 4 Medical School, School of Medicine, University of Patras, Patras, Greece

**Keywords:** COVID-19, mental health, who-5, GAD-7

## Abstract

**Introduction:**

COVID-19 pandemic and lockdown has brought a serious impact on physical and mental health.

**Objectives:**

The purpose of the present study was to estimate the impact of the first lockdown in Greece, on both quality of life and anxiety levels in different occupational groups.

**Methods:**

A cross-sectional on- line survey was conducted from 20^th^ of April to 4^th^ of May 2020. A 24-item anonymous questionnaire was administered to collect basic demographic and socioeconomic data. The 5-item WHO Well-Being Index (WHO-5, 0-100%, cut-off 52%) and the Generalized Anxiety Disorder Assessment (GAD-7) tools were used to assess well-being and anxiety, respectively. Statistical analysis was performed with SPSS for Windows v.24.0 Statistical Package.

**Results:**

A total of 575 participated in the study, 62.8% females, 48.5% aged between 40 to 59 years. 32.5% were employed in education sector, 32.5% in health sector and 20.3% as season workers in tourism sector. Males showed slightly higher levels of wellbeing (52.1 vs. 47.3, p=0.023) and lower levels of anxiety (7.1 vs. 8.2, p=0.023) compared to females. Factors associated with higher wellbeing and lower anxiety were higher education and income level, optimism, taking less protection measures, and being seasonal worker. Furthermore, participants with comorbidities and symptoms like headache, musculoskeletal pain, as well as feeling depressed or stressed revealed lower wellbeing and higher anxiety scores.

**Conclusions:**

Our study revealed an overall poor wellbeing and mild to moderate levels of anxiety during the lockdown. Actions should be taken to address and to prevent its serious impact on mental health.

**Disclosure:**

No significant relationships.

